# Tourist value co-creation behavior in immersive cultural tourism spaces: a case study of “the longest day in chang’an”

**DOI:** 10.3389/fpsyg.2026.1762355

**Published:** 2026-05-07

**Authors:** Jingwen Wang, Xiaoying Cao

**Affiliations:** 1 Yungang Study College, Shanxi Datong University, Datong, Shanxi, China; 2 Institute of Geographic Sciences and Natural Resources Research, Chinese Academy of Science, Beijing, China

**Keywords:** destination familiarity, emotional arousal, immersion, the longest day in Chang’an, tourist value co-creation behavior

## Abstract

**Introduction:**

Under the backdrop of continuous technological advancement and growing demands for tourist experiences, immersive cultural tourism spaces not only respond to the market need for profound engagement but also serve as crucial drivers for high-quality development in cultural industries.

**Methods:**

Taking “The Longest Day in Chang’an” as a case, this research constructs a “sense of immersion → emotional arousal → tourist value co-creation behavior” chain mediation model based on the Cognition-Affect-Behavior (CAB) framework and cognitive appraisal theory, with destination familiarity introduced as a moderating variable to elucidate the mechanisms of immersion’s impact on tourist value co-creation behavior. Partial Least Squares Structural Equation Modeling (PLS-SEM) was used for empirical analysis.

**Results:**

The findings reveal that: (1) Immersion exerts a significantly positive influence on emotional arousal; (2) Emotional arousal partially mediates the relationship between immersion and tourist participation behavior, while fully mediating the connection between immersion and tourist citizenship behavior; (3) Destination familiarity demonstrates a significant negative moderating effect on the “immersion → tourist citizenship behavior” pathway.

**Discussion:**

These conclusions provide theoretical insights for fostering tourist value co-creation behaviors and offer managerial implications for the governance of immersive cultural tourism spaces.

## 1 Introduction

As global consumption patterns evolve from acquiring physical goods to pursuing meaningful and memorable experiences, tourism has entered a new era characterized by experiential enrichment and emotional engagement (Blumenthal and Jensen, 2019; Lunardo and Ponsignon, 2020; Pine and Gilmore, 1999). The development of science and technology has become a key driving force for upgrading the tourism experience (Fan et al., 2022), and digital cultural tourism represented by the Metaverse has become an important trend in the development of the times (Jafar and Ahmad, 2023; Shamim et al., 2025). With the support and application of technology, intelligent cultural tourism immersive spaces have begun to emerge. Immersive cultural tourism spaces represent a novel consumption medium that combines technology, culture, art and tourism, providing tourists with deeply participatory and interactive experiences. These spaces emphasise immersive engagement, scenographic authenticity and emotional resonance, offering comprehensive, multisensory cultural experiences. Existing research on immersive tourism experiences predominantly focuses on virtual reality (Smith and Mulligan, 2021), augmented reality (Yim et al., 2017), and metaverse environments (Jafar and Ahmad, 2023), with most studies examining immersion formation mechanisms through external environmental factors such as authenticity, interactivity, and emotional resonance in virtual simulations (Zatori et al., 2018). At the same time, scholars are also concerned about the improvement of destination management through immersion (Li F. et al., 2023; Li et al., 2024; Trunfio et al., 2022). Nevertheless, immersive tourism is more than just a digital upgrade of traditional experiences; it represents a shift in the way tourists interact with cultural narratives and destinations. These experiences are often characterized by multisensory stimulation, narrative immersion, emotional resonance, and interactivity (Bec et al., 2019; Blumenthal and Jensen, 2019; Khater et al., 2025). Although existing research has explored the design features, user satisfaction, and technical implementation of immersive tourism products (Hudson et al., 2019; Shamim et al., 2025; Yawised and Apasrawirote, 2025), comparatively limited attention has been devoted to the internal psychological mechanisms through which immersive experiences transform tourists’ cognitive and emotional states and subsequently influence their behavioral responses. In tourism research, tourists’ value co-creation behaviors play an important role in enhancing interactive tourism experiences, and immersive experiences may further stimulate such behaviors (Li F. et al., 2023; Liu B. et al., 2023), their underlying mechanisms and boundary conditions remain inadequately elucidated. In particular, the emotional transformation processes that link immersive environments to tourist value co-creation behaviors remain insufficiently explored.

Based on the above, this study focuses on the emotional transformation of tourists in immersive cultural tourism spaces, exploring how immersive environments affect tourists’ experience processes and thus promote deeper levels of participation. The cognition-affect-behavior (C-A-B) framework provides a theoretical foundation for understanding consumer decision-making behavior, while cognitive appraisal theory offers a deeper explanation of how emotional responses arise from individuals’ cognitive evaluations of environmental stimuli. To address the limited attention given to the relationship between immersion and value co-creation in tourism research, this study integrates the Cognition–Affect–Behavior (CAB) framework with cognitive appraisal theory to construct and empirically validate the causal chain of “immersion → emotional arousal → value co-creation behavior”, explaining how immersion triggers emotional arousal and subsequently stimulates tourist value co-creation behaviors in immersive cultural tourism spaces. In doing so, this study hopes to enrich the theoretical understanding of value co-creation mechanisms within immersive tourism contexts, and provide practical and actionable strategies for innovation in the tourism industry, particularly in the design, governance, and experiential optimization of immersive cultural tourism spaces.

## 2 Literature review and hypotheses development

### 2.1 Theoretical background

#### 2.1.1 Cognition-affect-behavior framework

The cognition-affect-behavior (C-A-B) framework is that cognition (C) determines affect (A) which, in turn, results in behavior (B). This framework played a foundational role in the earliest systematic models of buyer behavior (Howard and Sheth, 1969). Subsequently, this method is implemented to elucidate consumer behavior and delineate the formation mechanism of individual behavior (Corstorphine, 2006). The term “cognition (C),” which is derived from personal experience (Deng et al., 2023), refers to the mental processes involved in acquiring, processing, storing, and using information. The term “affect (A)” is used to denote an individual’s emotions or feelings towards a specific object, constituting a subjective experience (Trabskaya et al., 2023). Behavior (B) is defined as the behavioral intentions or actual actions exhibited by an individual in response to internal or external stimuli (Kim et al., 2013), that is, the anticipated activities an individual plans to undertake related to a specific object or action (Smith, 2004). The CAB (Cognition-Affect-Behavior) framework and its subsequent refinements have proven particularly effective in analysing consumers’ responses to advertising, especially in revealing the mediating role of emotions in decision-making processes (Holbrook and Batra, 1987). Beyond the realm of advertising, this theoretical framework has been extensively applied to investigate the development of individual behaviors across a range of domains. These domains include shopping experiences (Fiore and Kim, 2007), online consumption (Chen and Lee, 2008), brand selection processes (Laroche et al., 1994), social networking sites (Kao and Lin, 2016), and museum experiences (Chen and Lee, 2008; Li et al., 2025). This framework provides significant insights into the affective and behavioral dimensions of consumer choice. In the domain of tourism and hospitality, it is frequently employed to assess consumer willingness and engagement. This further demonstrates its versatility in capturing the cognitive-emotional mechanisms that drive consumer behavior (Rahman et al., 2015). The Cognitive-Affective-Behavior (CAB) emphasizes cognitive and affective influences and their role in shaping behavior (Dweck and Leggett, 1988). In the context of tourism destination research, an interaction emerges between cognitive, emotional factors and behavioral intentions, which have considerable power to influence and shape behavior formation (Zheng et al., 2022).

This leads us to anticipate that when tourists enter technology-enhanced immersive cultural tourism spaces, their cognitive evaluation of immersion triggers emotional arousal, which in turn motivates them to perform value co-creation behaviors such as participation, assistance, feedback, and recommendation. Immersive tourism experiences are characterized by multi-sensory stimulation, spatial narratives, real-time interaction, and physical-digital hybrid environments, enabling visitors to transcend passive observation and engage more actively in cultural narratives and spatial symbols (Pine and Gilmore, 1999; Tussyadiah et al., 2018). Such environments, whether achieved through virtual/augmented reality, dramatic design, or gamified participation, cultivate a sense of presence and psychological engagement, thereby enhancing emotional resonance and social connection (Blumenthal and Jensen, 2019). Research indicates that immersive experiences not only enhance perceived authenticity and satisfaction but also increase the likelihood of prosocial and co-creative behaviors by strengthening visitors’ emotional and identity-based attachment to destinations (Blumenthal and Jensen, 2019). In this sense, immersive tourism spaces serve as emotional infrastructure, activating and sustaining visitor engagement at both the individual and collective levels, thereby laying the psychological foundation for meaningful value co-creation processes.

#### 2.1.2 Cognitive appraisal theory

In the early days of psychological study, the prevailing theory was that external stimuli directly determined individual emotions (Lange, 1885; Plutchik, 1965). However, this perspective fails to explain emotional variations among individuals under identical stimuli. Arnold (1960) posited that emotions do not directly emanate from external stimuli, but rather, they stem from an individual’s subjective appraisal of these stimuli. Lazarus (1982) also posits that emotions are not spontaneous; rather, they are the result of individuals’ cognitive assessments of the significance of external stimuli. Based on this, Lazarus (1984) developed the cognitive appraisal theory, which posits that emotions are activated by an individual’s cognitive evaluation of their environment. Specifically, divergent cognitive appraisals of the same situation, location or object can evoke distinct emotions, meaning that emotions are formed based on appraisal of events (Roseman, 2001). In subsequent research, scholars continue to enrich the connotation of cognitive appraisal theory. Appraisal regulates the action, feelings, behavior and responses (Moors et al., 2013; Scherer, 2019). Lazarus (1999) positions cognitive appraisal as the central mechanism linking environmental demands with personal meaning, emotion, and adaptation.

Cognitive appraisal has been further classified by scholars into diverse frameworks, including primary and secondary appraisals (Bagozzi et al., 1999), as well as dimensions such as goal congruence, goal relevance, certainty, coping potential, and responsibility attribution (Hosany, 2012). Among these, goal congruence and goal relevance have gained broad acceptance in the field of tourism research. The cognitive appraisal process constitutes an information-processing mechanism that serves as a causal determinant of emotions (Foroughi et al., 2022), subsequently influencing behavioral responses (Jiang, 2020). Cognitive appraisal theory has been extensively validated across a range of academic disciplines, including marketing (Foroughi et al., 2022), tourism studies (Jiang et al., 2020), and educational research (Cheng et al., 2019). Lazarus (1984) argue that they appraise and create meaning in a given situation which results in psychological processes and behavior. Lazarus (1991) emphasizes that cognition is indispensable to emotion, as it provides the evaluative basis through which individuals interpret environmental stimuli and assess their personal significance. Immersion is related to emotional engagement (Brown and Cairns, 2004; Jennett et al., 2008), which is key to creating memorable travel experiences (Kim, 2014). Memorable experiences, in turn, generate positive emotions, foster a desire to revisit, and increase the spread of positive word-of-mouth (Kim et al., 2010; Slåtten et al., 2011).

#### 2.1.3 Immersive and tourist value co-creation behavior

Carù and Cova (2013) argue that immersion is a process of accessing an experience through which consumers become fully absorbed in an experience by entering a themed and psychologically safe spatial enclave. Pine and Gilmore (1999) adopt an experience-economy perspective, defining immersion as “becoming physically (or virtually) a part of the experience itself”. Common to all these definitions is the idea that immersion is a subjective occurrence implying a spatio-temporal belonging in the world and is characterised by deep involvement in the present moment (Hansen and Mossberg, 2013). Because consumers are active co-producers of their own experiences, the ability to become immersed depends on their personal interpretations and emotional connections with the spatial context and narrative themes (Carù and Cova, 2015). While preliminary research on flow has chiefly examined subjective psychological states of immersion, technological advancements have since redefined the concept of immersion. This is now understood to signify “the capability to create interactive, realistic, and immersive environments through technology” (Schultze, 2010), characterised by emotionally charged engagement (Lindberg and Østergaard, 2015) and heightened affective responses (Hansen, 2014). This study focuses on the tourism experience of immersive cultural tourism space in a hybrid physical-digital environment, which is consistent with the technological connotation of immersive experience.

The product-dominant logic posits that firms create value by delivering products or technologies that fulfil customer needs (Vargo and Lusch, 2008). In contrast, the service-dominant logic highlights the collaborative value creation between service providers and customers (Grönroos and Gummerus, 2014), emphasising co-production and mutual benefit. In tourism studies, value co-creation behaviors are categorised into tourist participation behavior and tourist citizenship behavior, differentiated by their roles in service production. The former encompasses in-role actions such as pre-trip information gathering, co-production during travel, and social interactions (Teng and Tsai, 2020). The latter refers to extra-role, voluntary behaviors that support tourism enterprises, including experience sharing, feedback, and assisting others during or post-trip (Liu and Tsaur, 2014).

Research has demonstrated that elevated levels of immersion have the capacity to induce a sense of perceived pleasure, a form of perceived value (Zaman et al., 2010), which is a pivotal antecedent of value co-creation. Research has indicated that tourists who experience immersion exhibit higher levels of enthusiasm and motivation in co-creation activities (Nusenu et al., 2021). For instance, grounded theory analysis based on live tourism contexts demonstrates that immersion is a driving force for value co-creation (Li F. et al., 2023; Liu X. et al., 2023), while research on virtual reality (VR) attributes value co-creation behavior to immersive experiences (Royo-Vela et al., 2022; Mancuso et al., 2023). However, the research results lack empirical verification in physical-digital hybrid immersive spaces, and their mechanisms and boundary conditions have not been thoroughly explored. Thus, we propose that increased levels of immersion in immersive cultural tourism spaces have the capacity to enhance both the probability of tourist participation and the occurrence of citizenship behaviors. Therefore, we hypothesize that.

*H1*: Immersion has a significantly positive effect on tourist participation behavior.

*H2*: Immersion has a significantly positive effect on tourist citizenship behavior.

#### 2.1.4 Mediating role of emotional arousal

Dillman Carpentier and Potter (2007) have defined arousal as the intensity or level of an individual’s hedonic experience. The level of emotional arousal directly affects human behaviors, and the multiple components of tourism experience can trigger emotional arousal (Grönroos and Gummerus, 2014). It is characterised by the transition of an individual from a state of calmness to one of excitement, stimulation, concentration, and activity. This dimension is usually associated with the psychological and physiological excitement levels experienced in response to external stimuli (Russell and Pratt, 1980). The CAB framework provides theoretical support for understanding the relationship between “emotional arousal” and tourists’ cognition and behavior in immersive tourism experience, emphasizing the sequential path of cognitive evaluation (perceived immersion) triggering emotional response (emotional arousal), which in turn affects subsequent behavior (Zhang and Song, 2022). Cognitive appraisal theory posits that individuals form emotional responses based on their subjective cognitive evaluation of environmental stimuli (Choi and Choi, 2019). In the context of immersive tourism, tourists’ cognitive evaluation of the immersive environment is a critical factor in determining the type and intensity of their emotional response. Consequently, the cognitive appraisal theory lends further credence to the notion of an emotional bond between immersion and emotional arousal. This conclusion is further substantiated by the findings of previous studies conducted on immersive tourism. For instance, when tourists experience a heightened sense of immersion, they often exhibit a more positive emotional response, leading to increased emotional intensity and arousal (Li et al., 2015). Individuals who view highly immersive 3D animated films exhibit significantly elevated levels of emotional arousal (Visch et al., 2010). In the context of virtual tourism environments, immersion has been demonstrated to exert a substantial positive influence on emotional arousal (Song and Lu, 2024). In the context of immersive tourism, it has been observed that tourists’ evaluation of sensory-rich, technology-enhanced environments can elicit varying degrees of emotional arousal. Therefore, we hypothesize that.

*H3*: Immersion positively and significantly influences emotional arousal.

In accordance with the established characteristics of consumer behavior, it has been demonstrated that when consumers receive benefits from organisations, they are inclined to undertake actions to reward said organisations (Homans, 1958). In the context of tourism, the emotional experiences of tourists can be perceived as either beneficial or detrimental, which in turn evoke corresponding behavioral responses (Razzaq et al., 2017). It is generally accepted that positive emotional experiences have a beneficial impact on proactive behavior (Spector and Fox, 2002) and significantly enhances tourist participation (Zhao et al., 2024). In this study, emotional arousal, defined as a positive emotion, was found to be associated with tourists who were highly aroused and perceived benefits from destinations. These tourists were more inclined to engage in behaviors that involved the creation of value. The validity of this perspective is further substantiated by empirical studies in the field. Liu et al. (2021) identified positive effects of active emotions on tourist citizenship behavior. It was argued that high arousal levels create memorable experiences which in turn influence revisit intentions and recommendation behaviors (Huang and Bu, 2022), thereby affecting citizenship behaviors. Consequently, heightened emotional arousal has been demonstrated to be associated with an enhancement in participatory and citizenship behaviors. Therefore, we hypothesize that.

*H4*: Emotional arousal positively and significantly influences tourist participatory behavior;

*H4a*: Emotional arousal positively mediates the relationship between immersion and tourist participatory behavior;

*H5*: Emotional arousal positively and significantly influences tourist citizenship behavior;

*H5a*: Emotional arousal positively mediates the relationship between immersion and tourist citizenship behavior.

#### 2.1.5 Moderating role of destination familiarity

Familiarity is defined as consumers’ experience with a product or brand and their decision-related information (Alba and Hutchinson, 1987). Tan and Wu (2016) suggest that destination familiarity is defined as a multidimensional concept that includes both tourists’ subjective cognitive evaluation of the destination and the accumulation of information and experience. The extant literature suggests that tourists’ familiarity with a destination exerts a significant influence on their behavioral decisions (Gursoy and McCleary, 2004a). Once acquainted with a destination, tourists have been found to place greater reliance on internal information during the decision-making process, thereby reducing the necessity for external information (Gursoy and McCleary, 2004b). Consequently, less familiar tourists are more dependent on external information than highly familiar ones. As previously discussed, in immersive cultural tourism spaces, tourists’ perceived immersion has been shown to enhance their emotional arousal, thereby significantly increasing their engagement in value co-creation behaviors. However, it should be noted that these effects may be subject to boundary conditions, which may be a consequence of varying levels of destination familiarity. In more detail, highly familiar tourists rely on internal cognition for decision-making, thereby diminishing the role of external stimuli. Moreover, in the context of tourism, the familiarity has a detrimental effect on the relationship between the extremity of a tourism slogan and the attitudes of tourists towards said slogan (Zhang et al., 2017). This conclusion has been corroborated in psychological research (Xue et al., 2022). The present study posits that heightened destination familiarity serves to attenuate the impact of immersion on tourists’ value co-creation behaviors (tourist participatory behavior and tourist citizenship behavior). Therefore, we hypothesize that.

*H6*: Destination familiarity negatively moderates the effect of immersion on tourist participatory behavior;

*H7*: Destination familiarity negatively moderates the effect of immersion on tourist citizenship behavior.

### 2.2 Conceptual model construction

The C-A-B framework has inspired several theories such as the Consciousness—Emotion—Value model (Holbrook and Batra, 1987), affects event theory and appraisal theories of emotion (Lazarus, 1991; Roseman, 1984). This study constructs a research model based on the CAB (Cognitive Appraisal-Emotion-Behavior) theoretical framework, encompassing five core latent variables: immersion, emotional arousal, tourist participation behavior, tourist citizenship behavior, and destination familiarity. In terms of theoretical foundations, CAB theory emphasizes that individual behavior is the result of emotional responses triggered by cognitive appraisals of a situation. Based on this, this study proposes that the sense of immersion formed by tourists in immersive tourism contexts, as a form of cognitive appraisal, can significantly enhance their emotional arousal levels, thereby promoting more positive participation behavior and civic behavior.

**Figure 1:**
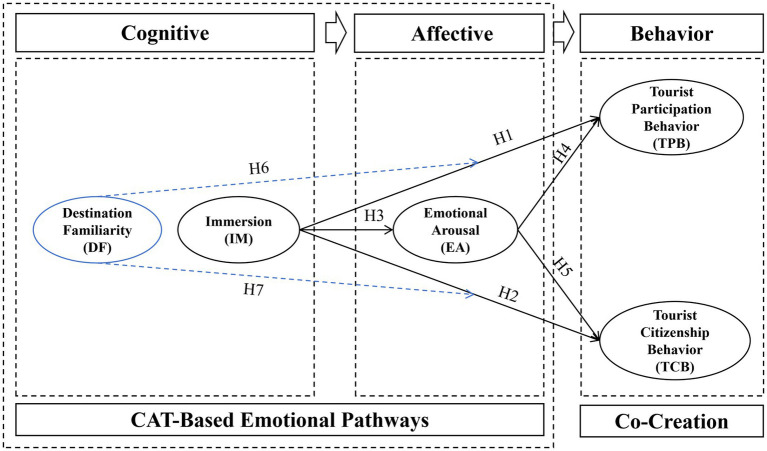
Research model.

To further unpack the cognitive-to-affective linkage, the cognitive appraisal theory (CAT) is introduced. Cognitive appraisal theory posits that emotions arise from individuals’ subjective evaluations of environmental stimuli (Arnold, 1960). Additionally, cognitive appraisal theory suggests that emotions arise from individuals’ subjective evaluations of environmental stimuli, providing a theoretical basis for the relationship between immersion and emotional arousal. Thus, the model further introduces a direct influence path of immersion on tourists’ value co-creation behavior, while examining the moderating role of destination familiarity in this mechanism to reveal its potential impact on the immersion effect. Finally, through hypothesis testing, the significance of the relationships among all paths in the model was validated, providing support for related theoretical and empirical research. This theoretical integration, which is multi-layered in nature, facilitates a more nuanced understanding of how perceived immersion leads to emotional arousal and subsequently drives value co-creation behaviors (Figure 1).

## 3 Research design and methods

### 3.1 Research design

**Figure 2:**
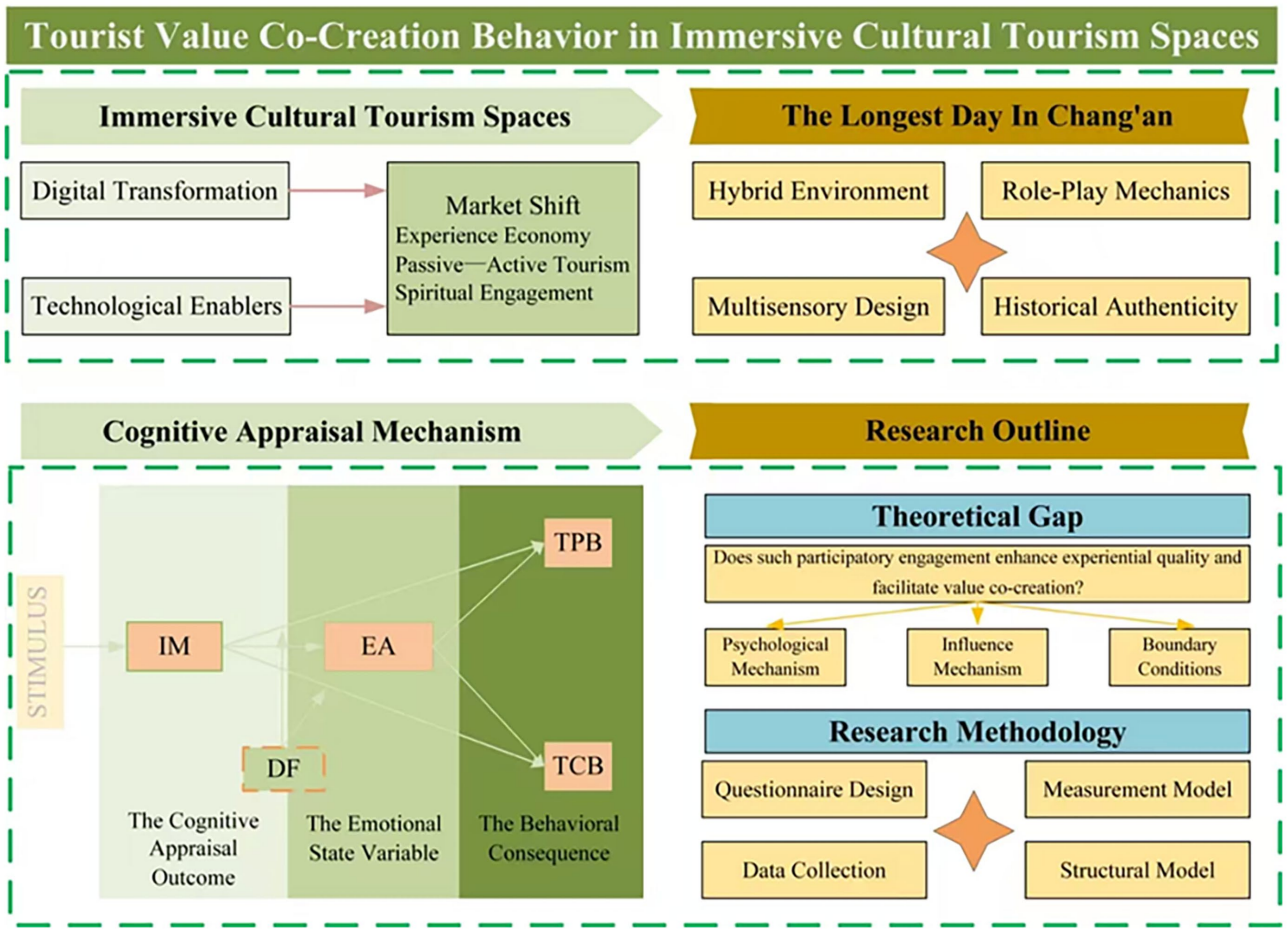
Design idea and research path.

The present study collected primary data through the administration of self-reported questionnaires and analysed the data using partial least squares structural equation modelling (PLS-SEM). PROCESS macro (Version 4) was employed to test the moderation effect. The questionnaire design was informed by well-established scales that have demonstrated high reliability and validity in previous domestic and international research. Minor adjustments were made to the wording of these scales to ensure respondents could understand the items intuitively and accurately. Specifically, the sense of immersion was measured using a 3-item scale from Jiang and Tu (2023). Emotional arousal was assessed with a 5-item scale adapted from Zhang et al. (2022). Tourist citizenship behavior was evaluated through a 10-item scale combining dimensions of helping, feedback, and recommendation based on Torres-Moraga et al. (2021) and Li et al. (2022). Tourist participation behavior (TPB) was measured using a 5-item scale from Gupta et al. (2023). Finally, destination familiarity was measured using a 3-item scale from Gursoy and McCleary (2004b). All items were measured on a 5-point Likert scale, with 1 representing “strongly disagree” and 5 representing “strongly agree” (Figure 2).

### 3.2 Study areas

**Figure 3:**
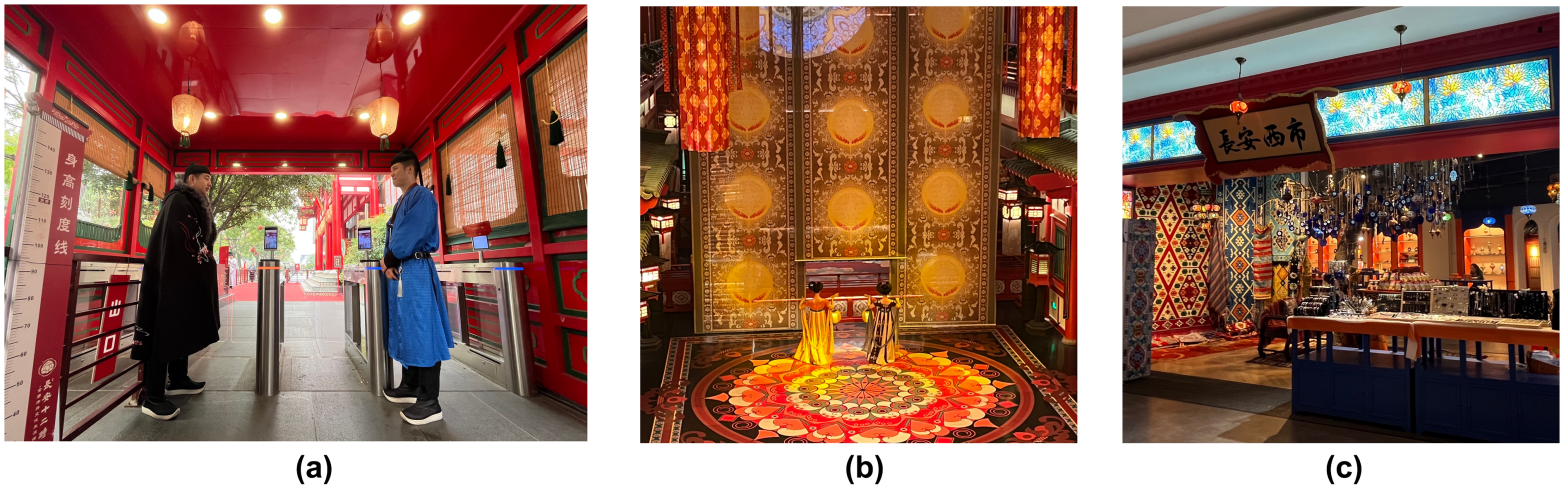
**(a)** Entrance; **(b)** Stage; **(c)** Chang’an street market.

**Figure 4:**
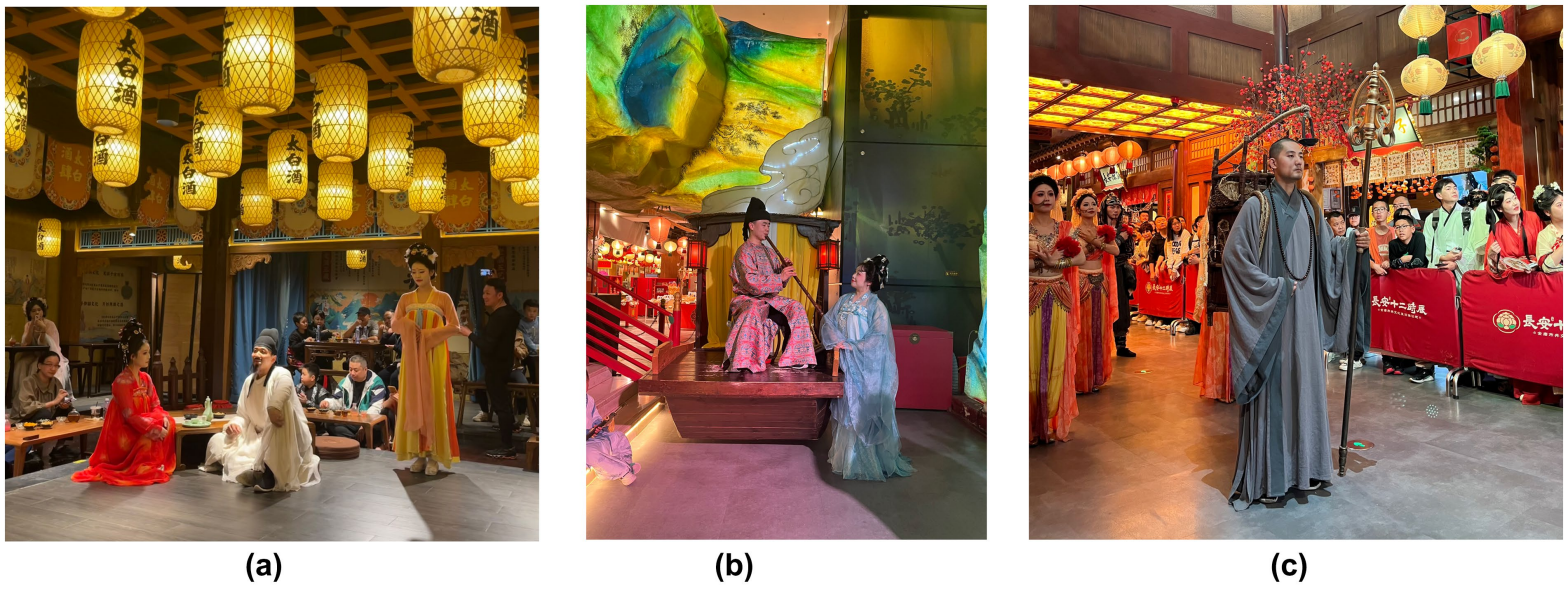
**(a)** Drinking with Li Bai; **(b)** Music interaction; **(c)** Character parade.

The Longest Day In Chang’an, located in Xi’an, Shaanxi Province, is China’s first immersive Tang Dynasty-themed attraction. It has become a representative example of immersive cultural tourism spaces due to its integration of multi-sensory technologies (e.g., digital lighting, spatial sound fields, scent devices) and blended virtual-physical scene construction (physical recreation of the film/TV IP The Longest Day In Chang’an). Visitors can immerse themselves in the Tang Dynasty by wearing Tang Dynasty costumes, watching Tang Dynasty performances, and tasting traditional cuisine, thereby experiencing a vivid sense of time displacement and cultural resonance with the prosperous Tang Dynasty era. This case exemplifies innovative tourism products and consumption scenarios, where traditional consumption behaviors are transformed into value co-creation practices (Figures 3, 4).

### 3.3 Data collection and analysis

This study employed a convenience sampling method, with an on-site questionnaire survey conducted at The Longest Day in Chang’an theme block from February 15 to 22, 2025. To mitigate potential representativeness bias, the survey covered both weekdays and weekends and was administered at different times of the day (morning, afternoon, and evening) to capture a diverse visitor flow. Questionnaires were distributed in multiple high-footfall areas, including entrances, exits, performance zones, and dining areas, which are typically where visitors spend extended periods. Since performances at the attraction run in cycles throughout the day, we confirmed whether respondents had fully experienced the core attractions before selection. Additionally, a systematic interception approach was adopted to reduce selection bias and ensure that respondents were not concentrated at a single time slot. Post-hoc statistical tests, including Harman’s single-factor test, were conducted to assess common method bias, and the results indicated no severe bias, supporting the robustness of the data.

*A priori* power analysis was conducted using G*Power 3.1 to determine the required sample size (Faul et al., 2009; Faul et al., 2007). Given that the primary analytical method is structural equation modeling, we based the calculation on the most complex regression equation within the model, which involved two predictors. With a medium effect size ƒ^2^ = 0.15, a significance level of *α* = 0.05, and a desired power of 0.80 (Cohen, 1988), the minimum required sample size was determined to be 68. A total of 310 questionnaires were distributed. After excluding responses with identical answers across all items or missing values, 284 valid questionnaires were retained, yielding a valid response rate of 91.61% (Table 1). This achieved sample size substantially exceeds both the minimum requirement derived from G*Power and the commonly recommended rule of thumb of 10 times the number of measurement items (Hair et al., 1998), thereby ensuring adequate statistical power for the proposed analyses.

**Table 1:** Respondents’ demographic information (*n =* 284).

Items	Categories	Percentage	Items	Categories	Percentage
Gender	Female	59.20%	Group size (self-inclusive)	One	9.50%
	Male	40.80%		Two	68.70%
Age	18 and below	7.40%		Three and more	21.80%
	19—30	66.50%	Professions	Corporate staff	19.70%
	31—50	23.90%		National civil servants	2.10%
	51—65	2.10%		Students	43.30%
	Over 65	0.00%		Staff in public institutions	12.00%
Visiting frequency	1 times	75.00%		Self-employed individuals/Freelancers	8.80%
	2 times	15.10%		Farmers	0.40%
	3 times	2.80%		Skilled workers	1.40%
	4 times and more	7.00%		Retirees	0.40%
Companies	Alone	6.70%		Unemployed/Laid-off	2.50%
	Families	39.80%		Homemakers	0.70%
	Friends or colleagues	48.60%		Others	8.80%
	Others	4.90%			

## 4 Results

### 4.1 Multicollinearity and common method bias analyses

In order to address the potential for multicollinearity interference in path analysis, this study utilised variance inflation factor (VIF) diagnostics. The analysis of the data yielded VIF values ranging from 1.000 to 3.890 for both the inner and outer models. This finding, which is strictly below the threshold of 5, confirmed the absence of multicollinearity (Ketchen, 2013). To mitigate the potential for common method bias in questionnaire surveys, Harman’s single-factor test was applied (Podsakoff et al., 2003). All measurement items of the key constructs were entered into an unrotated exploratory factor analysis with a fixed factor of one. The results indicated that a single factor accounted for 37.350% of the total variance, which is well below the recommended threshold of 50%. Therefore, common method bias is unlikely to be a serious concern in this study.

### 4.2 Measurement model analysis

This study validated the measurement model through composite reliability criteria: all latent variables exhibited factor loadings >0.6, with Cronbach’s alpha and composite reliability (CR) values >0.7 (Bagozzi and Yi, 1988), indicating robust internal consistency and confirming acceptable measurement reliability. The final measurement model included 25 items. Standardized factor loadings ranged from 0.612 to 0.897, Cronbach’s alpha values spanned 0.716 to 0.870, and CR values ranged from 0.837 to 0.909, satisfying reliability thresholds. The assessment of convergent validity, conducted through the utilisation of average variance extracted (AVE), yielded values ranging from 0.639 to 0.769 for all latent variables. This outcome surpasses the 0.5 benchmark (Fornell and Larcker, 1981), thereby substantiating robust convergent validity, as delineated in Table 2. During the evaluation of the measurement model, this study conducted item purification for each construct. Following recommendations in the existing literature (Anderson et al., 1988; Fornell and Larcker, 1981), we removed items with factor loadings below 0.6. Accordingly, one item from the emotional arousal scale was removed, namely “I feel awake”.

**Table 2:** Assessment of measurement model.

Variables	ID	Items	Standardized factor loading	Cronbach’s α	CR	AVE
Immersion (IM)	IM1	I was able to block out most other distractions	0.612	0.727	0.843	0.684
	IM2	I temporarily forgot the troubles of daily life	0.874			
	IM3	I was absorbed in tourist activities	0.897			
Emotional Arousal (EA)	EA1	I feel very excited	0.879	0.807	0.875	0.639
	EA2	I am very interested.	0.864			
	EA3	I feel stimulated	0.659			
	EA4	My attention is captured	0.777			
Destination Familiarity (DF)	DF1	I know a lot about The Longest Day in Chang’an	0.897	0.853	0.909	0.769
	DF2	I know more about The Longest Day in Chang’an than others	0.858			
	DF3	I know more about The Longest Day in Chang’an than my friends around me. Recommendation behavior	0.876			
Tourist Citizenship Behavior (TCB)	TCB1	Recommendation	0.820	0.716	0.837	0.631
	TCB2	Help	0.786			
	TCB3	Feedback	0.776			
Tourist Participation Behavior (TPB)	TPB1	During the visit, I am willing to spend time expressing my needs to staff	0.814	0.870	0.906	0.659
	TPB2	During the visit, I am willing to put effort into expressing my needs to staff	0.847			
	TPB3	To improve service quality, I am willing to provide suggestions to staff	0.744			
	TPB4	Throughout the service process, I am highly engaged	0.820			
	TPB5	When choosing service options, I am willing to participate in deciding	0.832			

The present study evaluated discriminant validity through three methods. Firstly, the Fornell-Larcker criterion (Fornell and Larcker, 1981) was applied by means of a systematic comparison of the square roots of the average variance extracted (AVE) for each latent variable against their inter-construct correlation coefficients. Empirical evidence has been provided to demonstrate that the square roots of the AVE for all latent variables significantly exceeded the absolute values of their corresponding correlations. As shown in Table 3, the data exhibited strong discriminant validity.

**Table 3:** Discriminant validity assessment.

Variable	IM	TPB	TCB	EA	DF
Immersion (IM)	**0.805**				
Tourist Participation Behavior (TPB)	0.474	**0.812**			
Tourist Citizenship Behavior (TCB)	0.495	0.611	**0.794**		
Emotional Arousal (EA)	0.732	0.489	0.591	**0.800**	
Destination Familiarity (DF)	0.051	0.036	0.279	0.098	**0.895**

Bold values on the diagonal are the square roots of the average variance extracted (AVE).

Secondly, discriminant validity was further verified using the cross-loading assessment method. As shown in Table 4, all observed variables exhibited considerably elevated loadings on their respective latent variables in comparison to cross-loadings with other constructs within the model, thereby rigorously adhering to the cross-loading discriminant criteria.

**Table 4:** Cross-loadings table.

Variable	TCB	IM	TPB	EA	DF
TCB1	**0.786**	0.277	0.456	0.347	0.239
TCB2	**0.776**	0.378	0.626	0.432	0.169
TCB3	**0.820**	0.482	0.405	0.508	0.251
IM1	0.234	**0.612**	0.259	0.338	0.072
IM2	0.414	**0.874**	0.418	0.628	0.022
IM3	0.494	**0.897**	0.438	0.721	0.044
TPB1	0.525	0.381	**0.814**	0.379	0.104
TPB2	0.482	0.338	**0.847**	0.380	0.074
TPB3	0.489	0.283	**0.744**	0.317	−0.015
TPB4	0.494	0.457	**0.820**	0.458	−0.025
TPB5	0.497	0.430	**0.832**	0.422	0.013
EA1	0.491	0.714	0.441	**0.879**	0.047
EA2	0.560	0.665	0.410	**0.864**	0.136
EA3	0.334	0.400	0.306	**0.659**	0.039
EA4	0.482	0.521	0.393	**0.777**	0.085
DF1	0.275	0.045	0.069	0.119	**0.897**
DF2	0.190	0.040	−0.045	0.047	**0.858**
DF3	0.252	0.048	0.045	0.078	**0.876**

Bold values indicate the factor loading of each item on its respective construct.

Thirdly, discriminant validity was assessed using the HTMT criterion. For reflective measurement models, discriminant validity is considered established when the HTMT values for all constructs fall below 0.9 (Gold et al., 2001). A bootstrapping procedure with 5,000 subsamples and the complete bootstrapping option was performed (Hair et al., 2018), and bias-corrected 95% confidence intervals were computed. A confidence interval containing the value of 1 would indicate a lack of discriminant validity (Henseler et al., 2015). Table 5 presents the HTMT values and their corresponding confidence intervals for each pair of constructs, with point estimates shown outside the parentheses and the bias-corrected and accelerated 95% confidence intervals provided within parentheses. The results indicate that, with the exception of the HTMT value between IM and EA, which is 0.902 and slightly exceeds the recommended threshold of 0.9, all other construct pairs have HTMT values below 0.9. Although the HTMT value for IM and EA slightly exceeds the threshold, its 95% confidence interval [0.841, 0.954] does not include 1. Overall, satisfactory discriminant validity among the constructs is confirmed.

**Table 5:** Results of HTMT.

Constructs	IM	TPB	TCB	EA
Immersion (IM)	–			
Tourist Participation Behavior (TPB)	0.570 [0.449; 0.674]			
Tourist Citizenship Behavior (TCB)	0.629 [0.514; 0.740]	0.788 [0.674; 0.884]		
Emotional Arousal (EA)	0.902 [0.841; 0.954]	0.572 [0.463; 0.661]	0.741 [0.649; 0.826]	
Destination Familiarity (DF)	0.072 [0.028; 0.099]	0.091 [0.046; 0.107]	0.343 [0.232; 0.448]	0.114 [0.058; 0.180]

### 4.3 Structural model analysis

The PLS-SEM approach necessitates the evaluation of two key metrics: the model’s coefficient of determination (R^2^) and the goodness-of-fit (GOF) index. The R^2^ values for emotional arousal (0.268), tourist citizenship behavior (0.358), and tourist participatory behavior (0.541) all exceeded the 0.10 threshold (Chin et al., 2008), indicating satisfactory explanatory power. The overall model’s GOF value was 0.454, which is higher than the 0.360 benchmark (Wetzels et al., 2009) and confirms a good model fit.

**Figure 5:**
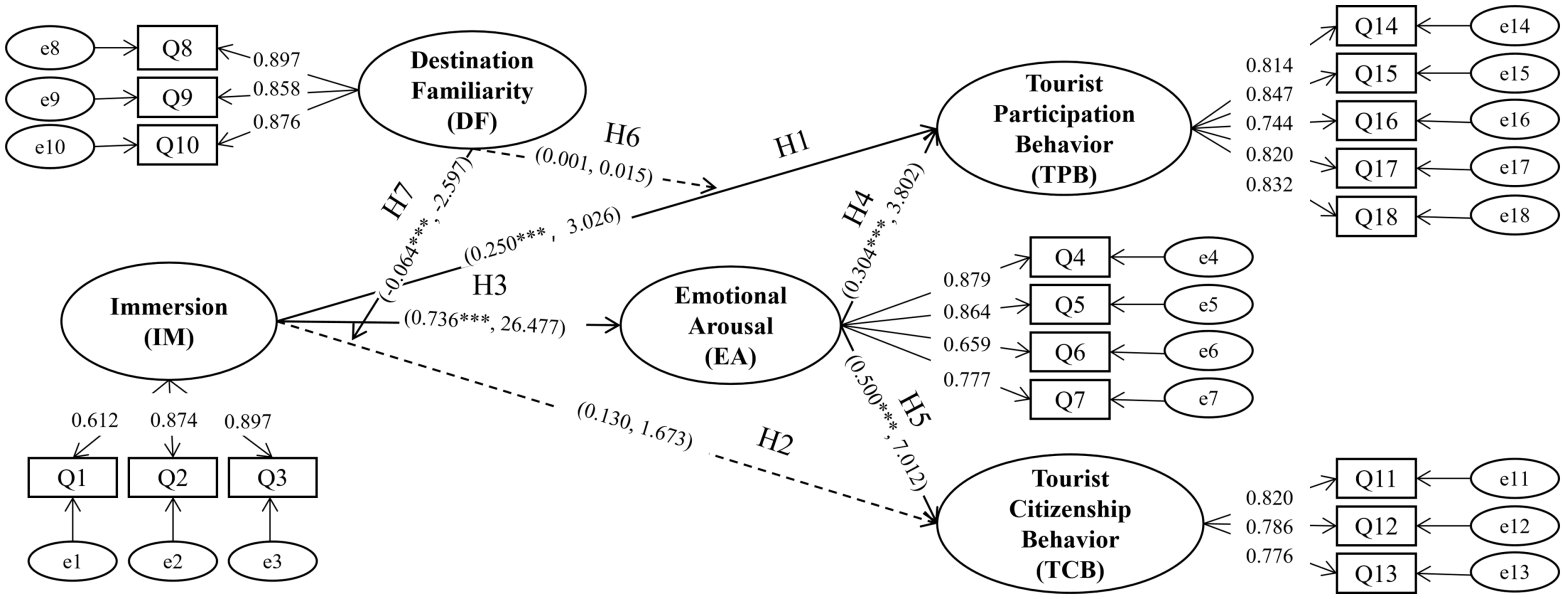
Research model results.

The results of the hypothesis testing are presented in Table 6 and Figure 5. The sense of immersion had a significant positive effect on tourist participatory behavior (*β* = 0.250, t = 3.026, *p <* 0.05), but no significant impact on tourist citizenship behavior (*β* = 0.130, t = 1.673, *p* > 0.05), thereby supporting Hypothesis 1 while rejecting Hypothesis 2. The findings indicated that the level of immersion experienced significantly enhanced emotional arousal (*β* = 0.736, t = 26.477, *p <* 0.001). Furthermore, the level of emotional arousal positively influenced both tourist participatory behavior (*β* = 0.304, t = 3.802, *p <* 0.001) and tourist citizenship behavior (*β* = 0.500, t = 7.012, *p <* 0.001). This validates H3, H4 and H5.

**Table 6:** Results of structure model.

Hypothesis	Path relationships	Path coefficient	T statistics	*p*-values	Supported
H1	IM → TPB	0.250	3.026	0.002	Yes
H2	IM → TCB	0.130	1.673	0.094	No
H3	IM → EA	0.736	26.477	0.000	Yes
H4	EA → TPB	0.304	3.802	0.000	Yes
H5	EA → TCB	0.500	7.012	0.000	Yes

### 4.4 Analysis of mediating effect

The present study employed the bootstrapping method, with 5,000 resamples at a 95% confidence interval, in order to test mediation effects (Table 7). Initially, in the “immersion→emotional arousal→tourist participatory behavior” path, both the direct effect (immersion on participatory behavior) and the total effect were found to be statistically significant. The mediating effect of emotional arousal (*β* = 0.224, t = 3.685, *p <* 0.001) was also significant, indicating partial mediation by emotional arousal between immersion and participatory behavior, thereby supporting hypothesis H4a.

**Table 7:** Results of mediating effect.

Effect	Path relationships	Path coefficient	T statistics	95% confidence interval	*P-*values
Direct effect	IM → TPB	0.250	3.026	[0.084,0.410]	0.002
	IM → TCB	0.130	1.673	[−0.025, 0.280]	0.094
Indirect effect	IM → EA → TPB	0.224	3.685	[0.109, 0.346]	0.000
	IM → EA → TCB	0.368	6.644	[0.264, 0.483]	0.000
Total effect	IM → TPB	0.474	8.689	[0.368, 0.585]	0.000
	IM → TCB	0.498	10.038	[0.398, 0.592]	0.000

For the “immersion→emotional arousal→tourist citizenship behavior” path, the indirect effect of emotional arousal was 0.368, with a 95% confidence interval ranging from 0.264 to 0.438, excluding zero, *p <* 0.001, confirming significant mediation. However, the direct effect of immersion on citizenship behavior was nonsignificant, while the total effect remained significant. This finding lends further support to Hypothesis H5a, which posits a direct correlation between emotional arousal and citizenship behavior in the context of immersion.

### 4.5 Analysis of moderating effects

The present study examined the moderating effects of destination familiarity, utilising the PROCESS 4.0 statistical software. The results of this analysis are presented in Table 8. The interaction term “immersion×destination familiarity” exerted a non-significant effect on tourist participatory behavior (*β* = 0.001, t = 0.015, p > 0.05), but a significant negative effect on tourist citizenship behavior (*β* = −0.064, t = −2.597, *p <* 0.05). Consequently, destination familiarity did not moderate the relationship between immersion and tourist participatory behavior (rejecting H6), while it negatively moderated the effect of immersion on tourist citizenship behavior (supporting H7). Therefore, H7 was confirmed, while H6 was rejected.

**Table 8:** Analysis of moderating effects.

Hypothesis	Path relationships	Path coefficient	T statistics	95% confidence interval	*P-ftable 3* values	Supported
H6	IM × DF → TPB	0.001	0.015	[−0.070, 0.071]	0.988	No
H7	IM × DF → TCB	−0.064	−2.597	[−0.112, − 0.015]	0.010	Yes

**Figure 6:**
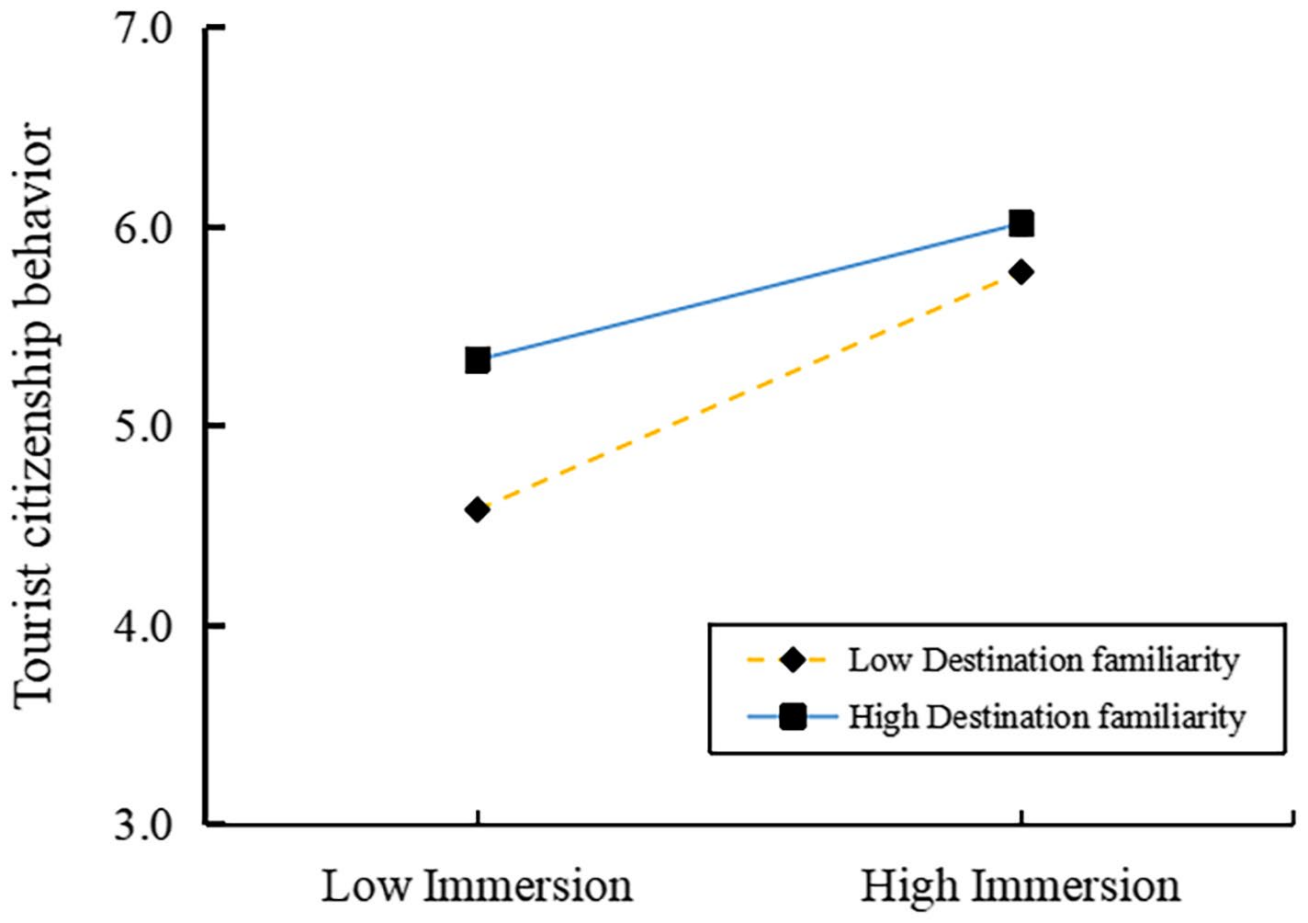
Simple slope plot.

To provide further validation of Hypothesis H7, this study conducted a simple slope test. The results indicated that immersion exerted a significant positive influence on tourist citizenship behavior, irrespective of destination familiarity levels. This observation was substantiated by the analysis, which yielded a simple slope value of 0.265 and a *p*-value of <0.001 for the high destination familiarity group. A similar outcome was observed in the low destination familiarity group, where the simple slope value was 0.463 and the p-value was also <0.001. However, the coefficient manifested greater strength in low familiarity groups compared to high familiarity groups, thereby indicating that heightened destination familiarity weakens the positive predictive effect of immersion on tourist citizenship behavior. This finding aligns with Hypothesis H7, as demonstrated in Figure 6.

## 5 Discussion

### 5.1 Immersion exerts a significant positive impact on tourists’ emotional arousal

By integrating the explanatory pathways of the CAB framework and cognitive appraisal theory regarding emotional generation mechanisms, the study reveals the multidimensional roles of dynamic psychological mechanisms in immersive tourism experiences, particularly the bridging role of emotional arousal in connecting immersion and tourist behavior (Li et al., 2025). Empirical results validate the applicability of the cognitive-emotional pathway in an immersive cultural tourism context, indicating that the stronger the sense of immersion, the more likely visitors are to experience emotional arousal. This finding not only supports previous theoretical understandings of the emotional value of immersive experiences but also validates that, within the context of immersive cultural tourism, individuals still follow the psychological response chain of “cognition → emotion → behavior” as outlined in the CAB model. The findings also substantiate the importance of enhancing immersion to stimulate tourists’ value co-creation behaviors. In immersive cultural tourism contexts, environmental stimuli such as narrative storytelling, multisensory settings, and interactive technologies can enhance tourists’ cognitive engagement. During this process, emotional arousal stimulates tourists’ value co-creation behaviors (Royo-Vela et al., 2022), fostering deeper emotional engagement and a virtuous cycle of interaction within the tourism context (Jafar and Ahmad, 2023). Accordingly, these findings provide a theoretical basis for destination managers to design immersive environments that strategically integrate technological, narrative, and interactive elements to effectively stimulate tourists’ emotional responses and co-creation behaviors.

### 5.2 Emotional arousal as a mediator of tourist value co-creation

Immersion significantly positively influences tourist participation behavior but shows no significant effect on tourist citizenship behavior. Emotional arousal partially mediates the relationship between immersion and participation behavior, while fully mediating the connection between immersion and citizenship behavior. The findings are consistent with the cognitive–affect–behavior pathway outlined (Holbrook and Batra, 1987). Although immersion has been shown to exert a direct and significant positive effect on tourist participation behavior, its direct impact on citizenship behavior is not statistically significant in our study. The immersion exerts a primary influence on citizenship behavior, operating chiefly through its effect on emotional responses. These findings offer partial support for the proposition that “immersion directly and positively predicts tourist value co-creation behaviors” (Li F. et al., 2023; Liu X. et al., 2023), and indicate that different dimensions of value co-creation may be influenced by immersion through distinct psychological pathways. A plausible explanation for this phenomenon lies in the distinction between participation behavior and citizenship behavior. Citizenship behavior represents an extra-role conduct requiring higher commitment, which may necessitate deeper emotional engagement and cognitive appraisal for activation, in comparison to participation behavior. In immersive cultural tourism spaces, the integration of technological innovation and experiential design promotes the evolution from tourist participation behavior (TPB) to more advanced tourist citizenship behavior (TCB). It establishes a theoretical foundation for implementing “precision experience management” in immersive cultural tourism spaces.

### 5.3 Destination familiarity negatively moderates the relationship between immersion and tourist citizenship behavior

The positive effect of immersion on citizenship behavior is weakened by higher destination familiarity, indicating that experienced visitors demonstrate no increased propensity for citizenship conduct. This finding appears to contradict the widely observed positive moderating role of destination familiarity in tourism research (Horng et al., 2012; Liu B. et al., 2023; Qu et al., 2022), yet it corroborates the findings of Zhang et al. (2017). The findings indicate that highly familiar tourists appear to place greater reliance on internal cognitive during the decision-making process, thereby diminishing the influence of immersion, a construct shaped by external stimuli, in promoting citizenship behavior. This underscores the imperative for a harmonious integration of external experiential innovations with tourists’ pre-existing cognitive frameworks, thereby ensuring the efficacious stimulation of citizenship behaviors. This study found that destination familiarity has a negative moderating effect on the influence of immersive experiences on tourists’ value co-creation behavior, further enriching our understanding of the boundaries of immersive tourism experiences. The lower familiarity is often accompanied by higher novelty-seeking motivation, greater attention resource investment, and more pronounced emotional responses, thereby leading to more positive psychological and behavioral outcomes (Teng and Tsai, 2020). In immersive cultural tourism spaces, it is necessary to balance innovation in tourists’ cognition and experience and maintain a sense of novelty in order to achieve sustained stimulation of citizenship behaviors.

### 5.4 Practical implications

As immersion is a critical antecedent of value co-creation, immersive scenarios can catalyze the transformation of tourists’ roles from “passive recipients” to “active contributors” (Sthapit et al., 2024). Compared with purely digital experience environments, immersive cultural tourism spaces emphasize real-world interaction while integrating technological elements (Jiang and Tu, 2023). Therefore, enhancing immersive experiences in cultural tourism spaces requires a multifaceted approach. Technologically, digital tools (e.g., augmented reality, multisensory stimulation) can enhance hyperrealistic environments and interactive experiences. Narratively, strengthening “storytelling” through IP-based scene recreation (e.g., literature, films, games) can guide emotional engagement and cognitive resonance, transforming tourists from “participants” to “co-creators” (Teng and Tsai, 2020). Service-wise, optimizing costume rentals, makeup services, and spatial segregation between immersive and daily environments can heighten uniqueness and ritualistic immersion. For “The Longest Day In Chang’an”, while the current story design is very appealing, and the costumes and set design are quite in line with the Tang Dynasty style, there are still shortcomings in the application of technology. Investment in technology will inevitably increase the scenic area’s costs in the future, but it is a crucial foundation for improving the quality of the scenic area.

Given familiarity’s negative moderation, managers should innovate products to reduce over-familiarity while maintaining “mystery” in marketing. Destination marketers and managers should enhance the immersive experience to counteract homogenization from the perspective of creativity (Khater et al., 2025), such as immersive theater, escape rooms, and interactive exhibitions. At the same time, designers should leverage multisensory stimuli, participatory narratives, and emotionally resonant stories to foster novelty and exploration, thereby amplifying immersion’s impact on citizenship behavior. Retaining some “unresolved mysteries” or “scene blank spaces” in destination promotions can enhance tourists’ emotional expectations and amplify the emotional contrast effect of actual experiences. While mystery marketing can attract tourists’ curiosity and increase initial engagement, it should be used carefully to maintain an appropriate level of destination familiarity. Tourism managers are encouraged to adopt a staged information disclosure strategy, gradually revealing key elements of the experience to sustain curiosity while reducing uncertainty. For “The Longest Day In Chang’an”, even though it is now part of a closed space in the mall, which can inspire tourists to have extraordinary experiences and enhance their emotional involvement by creating a scene that is completely different from the outside world, which deserves attention in terms of space design and management. As the number of visitors continues to grow, managers should pay greater attention to the experiences of repeat visitors and sustain their curiosity. For first-time visitors, destination familiarity should not be overlooked; instead, effective guidance should be provided to ensure they can achieve efficient and high-quality experiences during their first visit.

As an increasing number of tourists devote more time to taking and sharing photos than ever before (Li, 2020), immersive cultural tourism spaces should place greater emphasis on designing photogenic scenes that encourage tourists to fully engage in photo-taking activities. These visually compelling environments not only enhance the immersive experience but also stimulate emotional connection and social sharing. Moreover, as more tourists become aware of the symbolic significance of tourism rituals during their visits (Shi et al., 2022), destination management organizations are encouraged to thoughtfully design meaningful ritual experiences. By enriching the content and enhancing the behavioral complexity of such rituals (Liu and Wei, 2020), tourism operators can further deepen tourists’ sense of immersion, emotional resonance, and long-lasting memory. “Longest Day In Chang’an” has already a popular destination for travel photography, but it inevitably shows signs of homogenization with other scenic spots. It should increase the availability of mobile, flexible attractions, constantly innovate, and create unique travel photography experiences. Tourists should be encouraged to bring their own Tang Dynasty costumes, and creative competitions should be held regularly. The interactive experience of immersive cultural tourism spaces can be enhanced by harnessing tourists’ value co-creation behaviors.

### 5.5 Limitations and directions for future research

This study has certain limitations that warrant further investigation in future research. While the current research focused on the mediating role of emotional arousal and the moderating influence of destination familiarity in the relationship between immersion and tourists’ value co-creation behaviors, it is likely that other psychological or contextual variables may also shape this process. For instance, individual differences such as personality traits, cultural background, prior travel experiences, or motivational orientation may play critical roles in influencing how immersion translates into behavioral outcomes. Future studies could incorporate these variables to build a more comprehensive and nuanced model that captures the complexity of tourist experiences in immersive environments. Meanwhile, in terms of “mystery marketing”, future studies could further examine tourist curiosity as a critical psychological driver linking destination familiarity and engagement, in particular, exploring the dynamic interplay between curiosity, emotional arousal, and behavioral responses across different tourism contexts. In addition, this study did not fully explore the diversity of tourist participation modes and how they might differentially affect the perceived quality of immersive experiences. Participation in co-creation can vary from passive observation to active engagement, and the depth and nature of this involvement may influence emotional and cognitive responses in distinct ways. Future research could examine these differentiated participation patterns through more refined classification frameworks and explore their impact on satisfaction, memorability, and long-term loyalty. Moreover, the current study was conducted within a specific cultural tourism context, which may limit the generalizability of findings across different types of tourism experiences, such as adventure tourism, wellness tourism, or virtual tourism. Employing a multi-context comparative approach in future research would help verify the robustness of the proposed mechanisms across varied experiential settings.

## Data Availability

The original contributions presented in the study are included in the article/supplementary material, further inquiries can be directed to the corresponding author.
